# Supplementary material for: Evolution of Potentially Actionable Genomic Alterations in Advanced Prostate Cancer: A Real-World Analysis of Serial Circulating Tumor DNA Testing
Source: Cancers (Basel). 2025 Sep 18;17(18):3048. doi: 10.3390/cancers17183048 (PMC12468080; doi:10.3390/cancers17183048)
Supplement: Supplementary file 1 [file cancers-17-03048-s001.zip › cancers-3858678-supplementary.pdf]

AKT1	***CDKN2A***	***HNF1A***	***MSH6***	***RAD51D***
ALK	***CHEK2***	*HRAS*	*MTOR*	*RAF1*
APC	*CTNNB1*	*IDH1*	*MYC*	*RB1*
AR	*DDR2*	*IDH2*	*NF1*	*RET*
ARAF	*EGFR*	*JAK2*	*NFE2L2*	*RHEB*
ARID1A	*ERBB2*	*JAK3*	*NOTCH1*	*RHOA*
ATM	*ESR1*	*KEAP1*	*NPM1*	*RIT1*
BRAF	*EZH2*	*KIT*	*NRAS*	*ROS1*
BRCA1	*FANCA*	*KRAS*	***NTRK1***	*SMAD4*
BRCA2	*FBXW7*	*MAP2K1*	***NTRK2***	*SMO*
CCND1	*FGFR1*	*MAP2K2*	*NTRK3*	*STK11*
CCND2	*FGFR2*	*MAPK1*	***PALB2***	*TERT*
CCNE1	*FGFR3*	*MAPK3*	*PDGFRA*	*TP53*
CDH1	*GATA3*	*MET*	*PIK3CA*	*TSC1*
CDK12	*GNA11*	*MLH1*	*PMS2*	*VHL*
CDK4	*GNAQ*	*MPL*	*PTEN*	
CDK6	*GNAS*	*MSH2*	*PTPN11*	

Table S1. List of 83 genes assessed in Guardant360. Single nucleotide variants and insertions/deletions assessed in all 83 genes, while copy number amplifications and fusions assessed in 19 and 11 genes, respectively. Genes listed in bold were reported in this cohort with on-label alterations.
